# Correlation between pre-extraction periodontal diagnosis and peri-implant microbiome for patients treated with implant-retained overdenture – A retrospective cohort pilot study

**DOI:** 10.1371/journal.pone.0325711

**Published:** 2025-07-14

**Authors:** Xiaoxi Cui, Acela A. Martinez Luna, Alex Gillone, Qiang Wu, Mahmoud Serag, Ramiro Mendonca Murata

**Affiliations:** 1 Department of General Dentistry, School of Dental Medicine, East Carolina University, North Carolina, United States of America; 2 Department of Surgical Sciences, School of Dental Medicine, East Carolina University, North Carolina, United States of America; 3 Department of Public Health, Brody School of Medicine, East Carolina University, North Carolina, United States of America; 4 Department of Foundational Sciences, School of Dental Medicine, East Carolina University, North Carolina, United States of America; Universidade Federal de Pelotas, BRAZIL

## Abstract

Limited clinical data exists on the peri-implant microbiome in edentulous patients with implant-retained overdentures (IRO). This study aimed to examine the peri-implant microbiome and its correlation with pre-treatment periodontal diagnosis. A total of twenty-five patients with 50 implants were included, with demographic information and pre-treatment periodontal diagnosis collected. Clinical measurements and subgingival plaque samples were obtained from each implant, followed by 16S rRNA gene-targeted sequencing. Peri-implant parameters and microbiome were analyzed in relation to gender, remaining teeth prior to treatment, IRO function time, implant system, bone graft at implant placement, and peri-implant diagnosis. The mean age of patients was 71 years, with pre-extraction diagnoses including clinical healthy gingiva on a reduced periodontium (4 patients), localized periodontitis (4 patients), generalized periodontitis (7 patients), and generalized Stage IV Grade C periodontitis (10 patients). Nineteen implants were diagnosed with peri-implant mucositis, while 31 were healthy. The predominant genera detected were *Streptococcus*, *Rothia*, *Veillonella*, *Actinomyces*, and *Schaalia*. No significant correlation was found between pre-treatment periodontal diagnosis and peri-implant diagnosis. The findings suggest that while pre-extraction periodontal diagnosis might influence the peri-implant subgingival microbiome in IRO patients, it may not correlate with the clinical peri-implant diagnosis.

## Introduction

Dental implants have become a standard and successful treatment for replacing missing teeth in patients worldwide. Concurrent with increased frequency of implant placement is the rise of microbial induced peri-implant diseases [1,2]. Peri-implantitis has been identified as the leading cause of implant failure after osseointegration [3]. A multitude of risk factors have been associated with the onset and progression of peri‐implantitis, but the pathogenic peri-implant microbiota has been identified as one of the major etiologic factors [4].

The composition of the peri-implant biofilm can differ significantly based on peri-implant diagnoses [5], and it can also exhibit variations due to factors like changes in oral hygiene habits, systemic diseases, smoking and alterations in the oral environment, including implant surface characteristics, and peri-implant anatomy [5–9]. Contradictory findings have been reported regarding the microbiota associated with peri-implantitis, and its similarity with the microbiota associated to periodontitis [10]. Little is known about the peri-implant microbiome in the edentulous patients with implant-retained overdentures (IROs). In fact, most previous clinical studies only recruited a small group of patients and have only focused on limited number of common periodontal pathogens [11–13]. More information about the peri-implant microbiota is needed, including cross-sectional data on the composition of the microbiome around implants that serve as abutments for overdentures.

Previous studies have shown that full-mouth extractions may not eliminate periodontal pathogens, which can persist for extended periods in the oral cavity of edentulous patients with a history of periodontitis. [14–17]. It is important to investigate the potential effect of a patient’s pre-extraction periodontal status on the peri-implant microbiota following rehabilitation with an IRO. Additionally, there is limited knowledge about the peri-implant microbiome in the edentulous patients with IROs, and its correlation with the patients’ periodontal condition before full mouth extractions. This study hypothesizes that the severity of pre-treatment periodontitis will correlate with a higher quantity and proportion of pathogens associated with peri-implant disease in the biofilm surrounding dental implants. The aims of this study were to use a high-throughput next generation sequencing technique to examine the peri-implant microbiome, and to measure peri-implant clinical parameters around implants used for IROs and investigate their correlation with the pre-treatment periodontal diagnosis.

## Materials and methods

### Study design

This retrospective cohort study was approved by East Carolina University (ECU) Institutional Review Board (UMCIRB 21–000148). Dental records of completely edentulous patients who were treated with intraosseous implants, locator abutments, and mandibular implant-retained overdentures at ECU School of Dental Medicine (SoDM) from January 1, 2013 to March 31, 2022 were reviewed. The data were accessed for research purposes between July 1, 2021, and August 31, 2023. Inclusion criteria were: (1) completely edentulous adult patients (≥ 18 years old) who were dentulous or partially edentulous at initial presentation; (2) patients treated with intraosseous implants, locator abutments and mandibular IROs, which functioned uneventfully for at least three months; and (3) no history of peri-implantitis surgical treatment. Patient’s records were excluded if they did not meet the inclusion criteria and if they additionally had the following conditions: (1) use of antimicrobials during the 3 months prior to the study visit; (2) uncontrolled systemic disease or metabolic disorders (e.g., human immunodeficiency virus or diabetes mellitus) and use of medications (e.g., high-dose steroid therapy, bone therapeutic levels of fluorides, or bisphosphonates) that are detrimental to soft tissue and/or bone healing; (3) malignant diseases or other diseases treated with radiotherapy or chemotherapeutic agents (chemo-therapy); (4) severe alcohol or recreational drug use; (5) pregnancy and lactation; or (6) heavy smoking (greater than or equal to 20 or more cigarettes a day).

In a one-way ANOVA, a total sample of 25 subjects equally distributed among four pre-treatment periodontal diagnosis groups achieves 82% power to detect differences in probing depth (mm) means, assuming a standard deviation of 0.11 between group means, using an F-test with a significance level of 0.05. The common within-group standard deviation of probing depth is assumed to be 0.15. Two patients who were treated with IROs but did not meet the inclusion criteria were examined for calibration purposes. Peri-implant subgingival microbiologic plaque samples were collected and tested from these two patients. The clinical examination and subgingival microbiologic sampling were conducted by one calibrated examiner (X.C.) in this study. A total of twenty-five patients met the inclusion criteria and were recruited for this study. All participants gave their informed consent prior to their inclusion in the study.

### Pre-treatment periodontal examination data collection

Patients’ pre-treatment periodontal charts and radiographic images were retrieved from axiUm Electronic Health Record (EHR) Software (Exan Corporation, Vancouver, Canada) and reviewed. All patients received care in pre-doctoral student clinics. During the initial comprehensive examination, each patient underwent baseline panoramic or full-mouth series radiographs. Most patients also had a complete periodontal chart recorded by dental students and verified by a licensed dentist. Each periodontal chart included the following clinical parameters: plaque, probing depth (PD), clinical attachment loss (CAL), bleeding on probing (BOP), suppuration, width of keratinized tissue, furcation involvement, and mobility. Pre-extraction periodontal diagnoses were assigned to each patient by two calibrated board-certified periodontists (A.M. and A.G.) after reviewing periodontal charts and radiographic bone loss (RBL). For patients who did not have a complete periodontal chart at the initial examination, clinical notes and radiographs were reviewed to assist in determining the periodontal diagnosis. In addition to periodontal data, participants’ demographic characteristics (age, gender, race) and IRO information (implants, placement date, implant system, prosthesis delivery date) were also collected.

### Clinical examination of implants

Clinical examination of implants was conducted at the Clinical Research Center of ECU SoDM following the removal of the mandibular IRO in all cases. The clinical examination of implants included the following assessments: modified plaque index (mPlI) [18]; probing depth (PD) [19]; bleeding on probing (BOP); suppuration; width of keratinized tissue [20]. Probing depth, BOP and mPlI were recorded at six sites (mesio-buccal, midbuccal, disto-buccal, and the respective lingual/palatal sites) around the implants using a sterile implant probe (Colorvue UNC-12; Hu-Friedy) inserted into the sulcus with a light force. Probe was kept as parallel as possible to the longitudinal axis of implant. Probing depth was measured as the distance between the mucosal margin and the most apically probable portion of the sulcus, in millimeters [19]. Effort was made to position the periodontal probe with an angle of <15 degrees to the axis of the implant to avoid overestimation of measurement. Bleeding on probing (BOP) was recorded as positive if it occurred within 30 seconds of probing. Width of keratinized tissue was measured from the mucosal margin to the mucogingival junction at the narrowest position around the mandibular implant on both buccal and lingual aspects. The mucogingival junction was identified by the rolling technique, wherein the mucosa is rolled until the non-movable portion of the attached keratinized tissue is seen [20]. All measurements were rounded up to the nearest millimeter. A periapical radiograph of each implant was taken to evaluate the peri-implant bone level and to aid peri-implant diagnosis.

### Subgingival microbiologic sampling

Subgingival plaque samples were obtained from mesio-buccal, midbuccal, disto-buccal, and midlingual sites of each implant by the calibrated researcher. Sampling of each implant was performed using a kit consisting of sterile absorbent paper points and 1.5-mL sterile Eppendorf tubes. Before subgingival plaque sampling, supragingival plaque was removed from implants using a sterile curette, without penetrating the peri-implant sulcus. A careful relative isolation was performed with the help of an assistant, using cotton rolls and aspiration to prevent saliva contamination. The sampling sites were gently dried with an air syringe, and, subsequently, paper points were inserted into the peri-implant sulcus for 30 seconds. The paper points obtained from four sites of each implant were pooled and placed into an Eppendorf tube with 200 μl of TE buffer, followed by vortexing for 30 seconds. Paper points were removed from the Eppendorf tube. Eppendorf tubes were kept on ice in the clinic and subsequently stored in −80°C lab freezers until the next step was performed.

### Sample processing and 16S rRNA gene-targeted sequencing

Subgingival plaque samples were sent to the Forsyth Oral Microbiome Core for DNA extraction. A modified DNA isolation protocol was used to lyse plaque samples with overnight incubation with Ready-Lyse™ Lysozyme Solution (Lucigen Corporation, Cat. No. R1802M) followed by using a MasterPure DNA Purification Kit (Lucigen Corporation, Cat. No. MCD85201). DNA samples were sent to Zymo Research (Irvine, CA) for sequencing (V1V3 region). The 16S rRNA gene-targeted sequencing was performed using the Quick-16S™ NGS library preparation kit (Zymo Research, Irvine, CA). The 16S primers were used to amplify V1V3 region of the 16S rRNA gene. The 16S primers used to amplify the V1V3 region of the 16S rRNA gene were (adapter sequences not included.): 27f (AGRGTTYGATYMTGGCTCAG, 20 bp) and 515r (TBACCGCGGCTGCTGGCAC, 19 bp). The sequencing library was prepared using an innovative library preparation process in which PCR reactions were performed in real-time PCR machines to control cycles and therefore prevent PCR chimera formation. The final PCR products were quantified with qPCR fluorescence readings and pooled together based on equal molarity. The final pooled library was cleaned up with Select-a-Size DNA Clean & Concentrator™ (Zymo Research, Irvine, CA), then quantified with TapeStation® and Qubit®. The final library was sequenced on Illumina® MiSeq™ with a v3 reagent kit (600 cycles). The sequencing was performed with > 10% PhiX spike-in. Raw 16S V1V3 amplicon sequences obtained from Zymo Research were quality-filtered, denoised, pair-end merged, and chimera removed with the DADA2 tool (version 1.12.1) [21]. Amplicon sequence variants (ASVs) generated by DADA2 were subjected to species level taxonomy assignment based on the approach developed by Al-Hebshi et. al. [22] against the FOMC Reference Sequence Set (https://microbiome.forsyth.org/ftp/refseq) that consists of 1,015 full-length 16S rRNA sequences from HOMD V15.22, 356 from MOMD V5.1, and 22,126 from NCBI, a total of 23,497 sequences. Species-level taxonomy assignment algorithm is available at https://microbiome.forsyth.org/algorithm.php. Altogether these sequences represent a total of 17,035 oral and non-oral microbial species. Specie-level read count tables were imported using R’s “phyloseq” package (version 1.28.0) [23] for downstream analyses. Alpha diversity was calculated in 3 measurements: 1) number of species (observed), 2) Shannon index, and 3) Simpson index using “phyloseq” package’s “plot_richness” function [23]. Alpha diversity significance tests were evaluated with QIIME2 (version 2020.11) “alpha-group-significance” function [4]. Beta diversity NMDS plots were generated with the “ordinate” function in “phyloseq” and beta diversity significance tests were evaluated with QIIME2 “beta-group-significance” function [24]. ANCOM-BC (Analysis of Compositions of Microbiomes with Bias Correction) was used to test the significance of differential abundance between test groups [25]. Linear discriminant analysis LDA Effect Size (LEfSe) (version 1.0.0) was used to plot the effect size of differentially abundant features [26]. The FOMC analysis pipeline software version information is available at https://microbiome.forsyth.org/software.php.

### Statistical analyses

Descriptive statistical analysis of participants’ demographic characteristics and clinical parameters was conducted. The peri-implant clinical parameters of the two implants from the same patients were averaged and compared among the following groups using Kruskal-Wallis: (a) gender; (b) number of remaining teeth prior to treatment; (c) IRO function time; (d) implant system; (e) bone graft at the time of implant placement; and (f) peri-implant diagnosis. The relationship between pre-treatment periodontal diagnosis and peri-implant diagnosis was analyzed by a Chi-square test of independence. For 16S rRNA datasets among the above comparison groups, species richness (alpha diversity) was compared using the Kruskal-Wallis H test. Principal coordinates analysis (PCoA) based on the Bray-Curtis dissimilarity matrix was used to visualize differences in the peri-implant subgingival microbiome among groups. Statistical significance of group comparisons was assessed using permutational multivariate analysis of variance (PERMANOVA) based on the first two principal components. The PERMANOVA test evaluates whether the centroids of groups differ significantly, with p-values generated through permutation testing. Bacterial species with significantly different relative abundance in each comparison group were compared using LEfSe (Linear Discriminant Analysis Effect Size) at the most detailed taxonomic level available. The p-values were corrected for multiple testing through False Discovery rate (FDR) adjustment [27]. Statistical software R 4.0.3 and the Metacoder package were used for data analysis. A significance level of 0.05 was implemented. The STROBE guidelines were used to ensure the reporting of this observational study.

## Results

### General information and pre-treatment periodontal diagnosis of participants

Twenty-five patients (12 female and 13 male), each with two mandibular implants, were recruited, for a total of 50 implants. The mean age of the patients was 71 years (standard deviation 6.1, range 59–83 years). The majority of patients (72%) were White, while four (16%) were Black or African American, and three (12%) were Hispanic or Latino. Additionally, 88% of patients were not current smokers, including five former smokers who had quit for at least one year [28]. Three patients—two male and one female—were current smokers. Among the five former smokers, four were male patients, all of whom had quit more than 10 years ago. The remaining former smoker was a female patient who had quit three years ago. Ten patients had less than 10 teeth remaining prior to full mouth extraction and IRO treatment, while 15 patients had equal to, or more than 10 teeth left prior to full mouth extraction. According to patients’ pre-treatment periodontal examination and radiographic examination, the patients were categorized into four groups based on the 2018 Classification of Periodontal Diseases [29]: four patients had clinical healthy gingiva on a reduced periodontium; four patients had localized periodontitis (Stage II to IV and Grade B or C); seven patients had generalized periodontitis but did not fall into the Stage IV Grade C category (Stage I to III, Grade A or B); ten patients had generalized Stage IV Grade C periodontitis. Since the majority of patients underwent full mouth extraction treatment right after the initial examination and did not receive periodontal treatment at ECU, there is no record indicating whether the four patients with clinical healthy gingiva on a reduced periodontium had a history of periodontitis.

Each patient’s pre-extraction probing depth range, clinical attachment loss range, periodontal diagnosis and the intervals between key events in the study are presented in Table 1. The key events include the time between the most recent extraction and implant placement, the interval between implant placement and IRO delivery, and the interval between IRO delivery and the research exam (IRO function time). Three (12%) patients had implants placed prior to the completion of full-mouth extraction, six (24%) underwent immediate implant placement, and sixteen (64%) had implants placed at least three months after extractions. The mean interval between implant placement and IRO delivery was approximately 9 months. The IRO functional period was defined as the time between IRO delivery and the research examination. Sixteen (64%) patients had worn their IRO for more than one year, while nine (36%) had worn it for less than a year.

**Table 1:** Pre-extraction periodontal conditions and the intervals between key events for each patient in the study.

Patient	Pre-treatment periodontal diagnosis	Pre-treatment PD range (mm)	Pre-treatment CAL range (mm)	Interval between the most recent extraction and implant placement (days)	Interval between implant placement and IRO delivery (days)	IRO function time (days)
1	Generalized periodontitis, Stage III Grade B	3–5	9–10	238	312	1926
2	Generalized periodontitis, Stage IV Grade C	2–7	1–7	0	183	768
3	Localized periodontitis, Stage IV Grade B	2–5	1–7	182	257	933
4	Generalized periodontitis, Stage IV Grade C	2–8	4–13	465	203	1017
5	Generalized periodontitis, Stage IV Grade C	1–4	2–10	0	409	387
6	Clinical gingival health on a reduced periodontium	Missing	Missing	0	301	409
7	Generalized periodontitis, Stage IV Grade C	Missing	Missing	174	110	3062
8	Generalized periodontitis, Stage IV Grade C	1–8	1–13	0	426	150
9	Generalized periodontitis, Stage II Grade B	1–5	Missing	0	156	1203
10	Generalized periodontitis, Stage II Grade B	2–4	2–7	476	147	1566
11	Localized periodontitis, Stage II Grade B	1–6	0–6	282	427	1846
12	Generalized periodontitis, Stage IV Grade C	2–10	0–11	155	290	370
13	Generalized periodontitis, Stage IV Grade C	1–10	2–13	193	387	505
14	Localized periodontitis, Stage IV Grade C	2–9	0–11	231	299	184
15	Clinical gingival health on a reduced periodontium	2–3	2–6	95	286	434
16	Generalized periodontitis, Stage III Grade B	1–6	2–11	259	250	176
17	Generalized periodontitis, Stage III Grade B	1–5	2–12	0	133	216
18	Clinical gingival health on a reduced periodontium	1–3	0–7	−658	249	638
19	Generalized periodontitis, Stage III Grade B	2–6	2–7	503	277	105
20	Clinical gingival health on a reduced periodontium	2–3	2–8	−254	515	238
21	Generalized periodontitis, Stage IV Grade C	1–7	1–11	1968	191	320
22	Generalized periodontitis, Stage IV Grade C	1–4	0–6	942	301	112
23	Localized periodontitis, Stage IV Grade B	1–6	1–7	652	235	309
24	Generalized periodontitis, Stage III Grade B	Missing	Missing	176	132	903
25	Generalized periodontitis, Stage IV Grade C	1–7	0–9	−322	339	1359
Mean				230.3	272.6	765.4
SD				482.1	104.9	725.1
Minimum				−658	110	105
Maximum				1968	515	3062

PD: probing depth; CAL: clinical attachment loss; IRO: implant-retained overdenture.

### Implant clinical examination and pre-treatment diagnosis correlation

For the 50 implants, 19 exhibited peri-implant mucositis (erythema, bleeding on gentle probing, swelling and/or suppuration), while 31 exhibited peri-implant health. None of the implant included in the study exhibited peri-implantitis (inflammation in the peri-implant mucosa and subsequent progressive loss of supporting bone) according to the 2018 Classification of Peri-implant Diseases [30]. Thirty-six implants were bone level tapered screw-vent implants with microtextured titanium surface, and 12 implants were bone level tapered implants with sand-blasted, large grit, acid- etched (SLA) surface. Only two implants were bone level tapered implants with micro-textured OSSEOTITE surface, they were not included in the statistical analysis due to extremely small sample size. Bone grafting at time of implant placement was completed in ten implants. The means and standard errors of implant clinical parameters were categorized based on the subgroups identified by each grouping variable (Table 2). The mean (±SE, standard error) PD of all implants was 1.56 (± 0.08) mm, mean BOP was 0.16 (± 0.04), mean width of keratinized tissue was 1.88 (± 0.30) mm on the buccal sites, and 1.40 (± 0.29) mm on the lingual sites. BOP and mPlI were significantly higher (P < 0.05) in the peri-implant mucositis group than in the peri-implant health group. Mean BOP in the peri-implant mucositis group was 0.40 (± 0.06), and mean mPlI was 1.41 (± 0.14); while mean BOP in peri-implant health group was 0.00 (± 0.00), and mean mPlI was 0.92 (± 0.14). The width of buccal keratinized tissue was significantly greater (P < .05) on bone level tapered implants with SLA surface than on bone level tapered screw-vent implants with microtextured titanium surface. Mean PD was significantly higher (P < .05) in male patients than in female patients; however, the difference was less than 0.5 mm. No significant differences were observed regarding number of remaining teeth prior to treatment, IRO function time, bone graft at the time of implant placement, and pre-treatment periodontal diagnosis. The relationship between pre-treatment periodontal diagnosis and peri-implant diagnosis was analyzed by a Chi-square test of independence and a repeated measures model and both showed no within-subject correlation (Table 3).

**Table 2:** Characteristics of patients, and comparisons of implant clinical parameters among groups.

Group	Category	Number (%)	PD (mm)	BOP	mPlI	Width of keratinized tissue – Buccal (mm)	Width of keratinized tissue – Lingual (mm)
Gender	Female	12 (48)	1.42 (0.11)^*^	0.13 (0.05)	1.04 (0.19)	1.88 (0.40)	1.08 (0.34)
	Male	13 (52)	1.70 (0.11)^*^	0.18 (0.07)	1.06 (0.18)	1.88 (0.45)	1.69 (0.46)
Number of teeth prior to treatment	< 10 teeth	10 (40)	1.47 (0.16)	0.09 (0.03)	0.98 (0.19)	1.50 (0.37)	1.05 (0.36)
	≥ 10 teeth	15 (60)	1.63 (0.09)	0.20 (0.06)	1.10 (0.18)	2.13 (0.43)	1.63 (0.42)
IRO function time	Less than 1 year	9 (36)	1.55 (0.12)	0.16 (0.08)	1.06 (0.17)	1.72 (0.57)	1.11 (0.43)
	More than 1 year	16 (64)	1.57 (0.11)	0.16 (0.05)	1.05 (0.18)	1.97 (0.35)	1.56 (0.38)
Implant system	Bone level tapered screw-vent implants with microtextured titanium surface	18 (75)	1.63 (0.10)	0.12 (0.04)	0.97 (0.17)	1.61 (0.35)^*^	1.50 (0.34)
	Bone level tapered implants with sand-blasted large grit acid-etched surface	6 (25)	1.36 (0.15)	0.28 (0.12)	1.24 (0.21)	3.00 (0.32)^*^	0.67 (0.40)
Bone graft at the time of implant placement	Yes	5 (20)	1.38 (0.11)	0.12 (0.06)	0.90 (0.28)	2.10 (0.53)	2.00 (0.67)
	No	20 (80)	1.61 (0.10)	0.17 (0.05)	1.09 (0.15)	1.83 (0.35)	1.25 (0.32)
Peri-implant diagnosis^†^	Peri-implant health	31 (62)	1.51 (0.09)	0.00 (0.00)^*^	0.92 (0.14)^*^	1.85 (0.36)	1.45 (0.36)
	Peri-implant mucositis	19 (38)	1.74 (0.14)	0.40 (0.06)^*^	1.41 (0.14)^*^	1.29 (0.38)	1.21 (0.37)
Pre-treatment periodontal diagnosis	Clinical gingival health on a reduced periodontium	4 (16)	1.71 (0.30)	0.23 (0.10)	1.25 (0.17)	2.00 (0.68)	0.75 (0.60)
	Localized periodontitis, Stage II to IV, Grade B or C	4 (16)	1.44 (0.10)	0.23 (0.18)	1.17 (0.42)	2.50 (0.94)	0.88 (0.31)
	Generalized periodontitis, Stage I to III, Grade A or B	7 (28)	1.46 (0.16)	0.06 (0.03)	1.01 (0.22)	1.64 (0.55)	1.07 (0.44)
	Generalized periodontitis, Stage IV Grade C	10 (40)	1.63 (0.12)	0.17 (0.06)	0.96 (0.25)	1.75 (0.50)	2.10 (0.56)

The peri-implant clinical parameters of the two implants from the same patients were averaged before calculating the means and standard errors. Kruskal-Wallis tests were used for statistical comparisons.

* p < 0.05

† Implant-level information. The peri-implant clinical parameters of the two implants from the same patients were not average if the two implants had different diagnosis.

PD: probing depth; BOP: bleeding on probing; mPlI: modified plaque index; IRO: implant-retained overdenture.

**Table 3:** Relationship between pre-treatment periodontal diagnosis and peri-implant diagnosis based on Chi-square test.

		Peri-implant diagnosis		
		Health	Mucositis	p
Pre-treatment periodontal diagnosis	Clinical gingival health on a reduced periodontium	3 (37.5)	5 (62.5)	0.237
	Localized periodontitis, Stage II to IV, Grade B or C	4 (50)	4 (50)	
	Generalized periodontitis, Stage I to III, Grade A or B	11 (78.6)	3 (21.4)	
	Generalized periodontitis, Stage IV Grade C	13 (65)	7 (35)	

### Microbiome analysis of peri-implant sites

The raw data of all libraries generated during this study is publicly available at the Sequence Read Archive (SRA) portal of NCBI under accession number BioProject PRJNA1190949. A total of 5056 unique merged and chimera-free ASV sequences were identified, and their corresponding read counts for each sample are available in the supporting information “ASV Read Count Table by Sample” with rows for the ASV sequences and columns for sample (S1 Table).

### Alpha diversity and microbial richness

**Fig 1:**
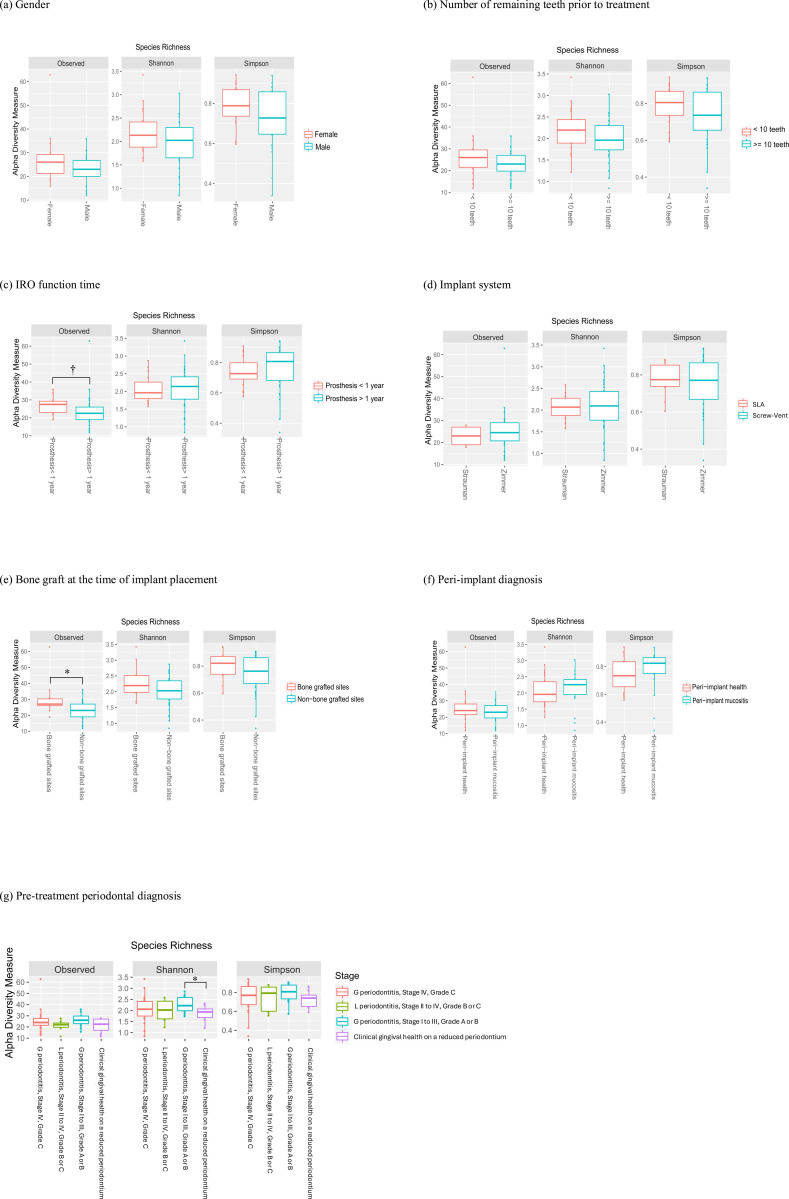
Alpha diversity of peri-implant subgingival microbiome among different comparison groups. IRO: implant-retained overdenture. SLA: sand-blasted, large grit, acid- etched. G periodontitis: generalized periodontitis. L periodontitis: localized periodontitis. *: p < 0.05. †: p < 0.01.

The analysis of the peri-implant microbiome revealed significant differences in bacterial species richness across various groups (Fig 1). Alpha diversity, measured by the Shannon diversity index, demonstrated that patients with generalized Stage I–III periodontitis exhibited greater bacterial diversity in peri-implant subgingival plaque compared to those with clinical healthy gingiva on a reduced periodontium. While the observed number of species appeared significantly lower in peri-implant sites with IROs functioning for over one year, Shannon and Simpson indices were slightly higher in this group. Additionally, sites with bone grafts at implant placement showed increased bacterial richness. However, no significant differences in alpha diversity were observed based on factors such as gender or the number of remaining teeth prior to treatment. To assess sequencing depth sufficiency and its potential impact on diversity estimates, alpha rarefaction curves based on Shannon, Simpson, and observed features metrics were generated. These rarefaction curves (included in Supporting Figures, S2–S4 Figure) show that the diversity metrics plateaued across samples, suggesting adequate sequencing depth for robust alpha diversity comparisons.

### Beta diversity and microbial composition

**Fig 2:**
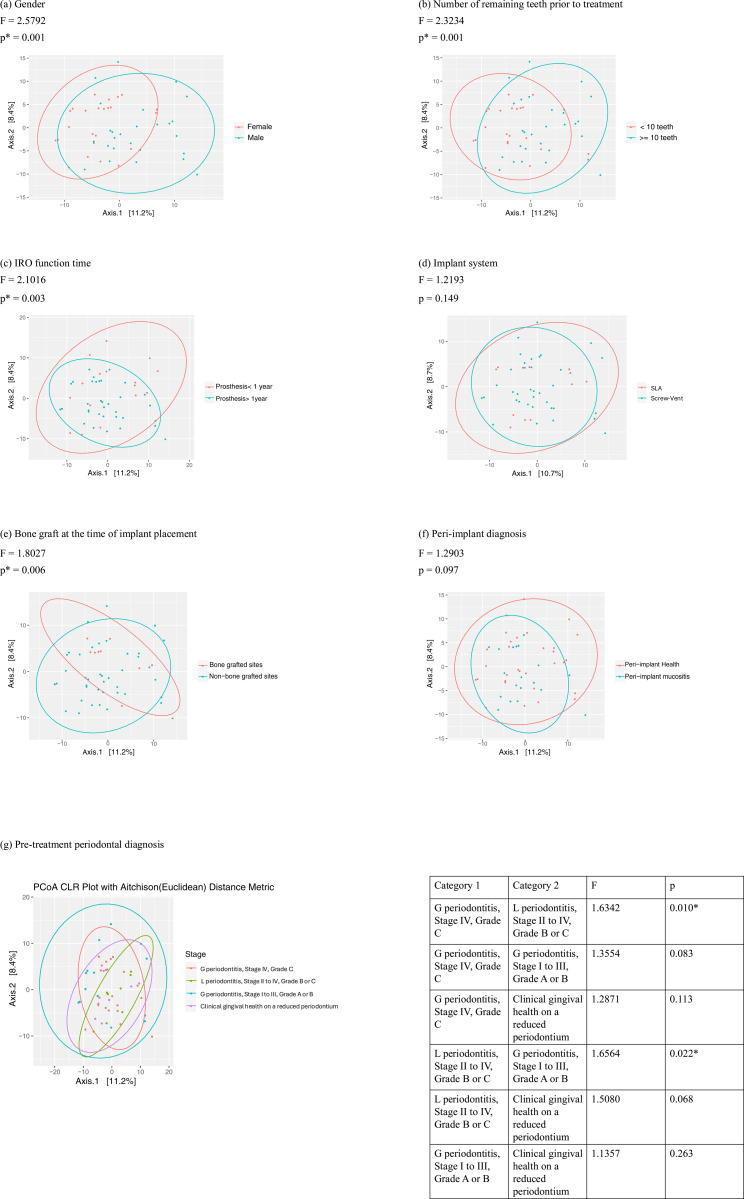
Principal coordinates analysis (PCoA) of peri-implant subgingival microbiome among different comparison groups. IRO: implant-retained overdenture. SLA: sand-blasted, large grit, acid- etched. G periodontitis: generalized periodontitis. L periodontitis: localized periodontitis. *: p < 0.05.

Beta diversity analysis provided further insights into the variation in microbial composition among groups. Principal coordinates analysis (PCoA) revealed distinct clustering of peri-implant microbial communities based on pre-treatment periodontal diagnosis, IRO function time, and the presence of bone grafts (Fig 2). These differences were statistically significant, as confirmed by permutational multivariate analysis of variance (PERMANOVA, p < 0.05). The clustering patterns suggest that these factors significantly influence the composition of peri-implant microbial communities.

### Taxonomic profiles of peri-implant microbiomes

**Fig 3:**
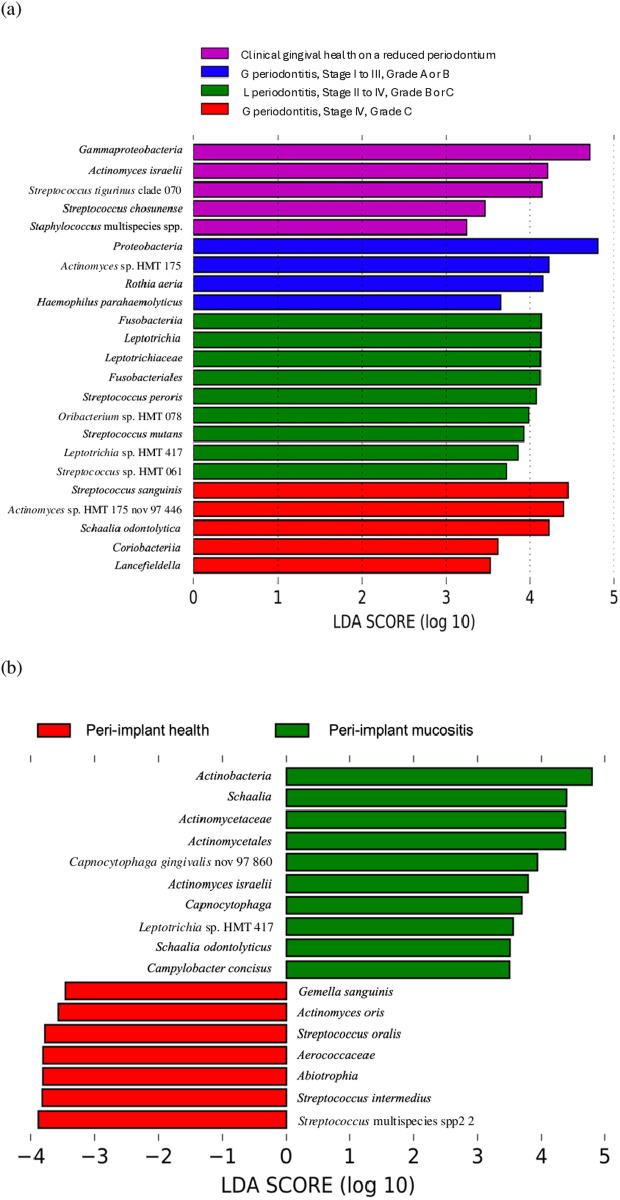
Differential species identified associated with different groups using Linear Discriminant Analysis Effect Size (LEfSe) method. (a) Differential species identified in different pre-treatment periodontal diagnosis groups. (b) Differential species identified in different peri-implant diagnosis groups. G periodontitis: generalized periodontitis. L periodontitis: localized periodontitis.

Taxonomic representation of statistically consistent differences among different pre-treatment periodontal diagnosis groups and between different peri-implant diagnosis groups was performed using the linear discriminant analysis effect size (LEfSe) method (Fig 3). At the genus level, dominant bacteria identified in peri-implant subgingival plaque across all groups included *Streptococcus*, *Rothia*, *Veillonella*, *Actinomyces*, and *Schaalia*. However, distinct bacteria were associated with specific pre-treatment periodontal diagnoses [Fig 3(a)]. Patients with clinical healthy gingiva on a reduced periodontium exhibited an enrichment of *Streptococcus tigurinus*, *Actinomyces israelii*, *Streptococcus chosunense*, *Staphylococcus* multispecies spp. and bacteria within the Gammaproteobacteria class. In contrast, localized periodontitis was characterized by the presence of nine bacterial species, including five from the Fusobacteriales order and two from the *Streptococcus* genus. For generalized Stage I–III periodontitis, increased abundances of *Proteobacteria*, *Actinomyces* sp. HMT 175, *Rothia aeria*, and *Haemophilus parahaemolyticus* were observed. Additionally, generalized Stage IV Grade C periodontitis was associated with an enrichment of *Streptococcus sanguinis*, *Actinomyces* sp. HMT 175, *Schaalia odontolytica*, *Atopobiaceae Lancefieldella*, and bacteria from Coriobacteriia class. At the phylum level, Firmicutes and Proteobacteria were the most relatively abundant phyla detected in the clinically healthy gingiva on a reduced periodontium group. Firmicutes and Fusobacteria were more abundant in the localized periodontitis group. In the generalized periodontitis groups, the relatively abundant phyla shifted to Actinobacteria and Proteobacteria.

Distinct microbial profiles were also observed between peri-implant health and mucositis sites [Fig 3(b)]. Three *Streptococcus* species and two species in the family *Aerococcaceae* were among the seven representative bacteria from plaque collected from peri-implant health implants. Among them, *Gemella sanguinis*, *Actinomyces oris*, and *Streptococcus intermedius* were not detected at all from peri-implant mucositis sites, and only a minimal amount of *Streptococcus oralis* was detected from peri-implant mucositis sites. In contrast, peri-implant mucositis sties exhibited unique bacterial signatures. Ten bacteria were associated with peri-implant mucositis. Among them, *Capnocytophaga gingivalis* and *Leptotrichia* sp. HMT 417 were only detected from peri-implant mucositis sites, and only a minimal amount of *Schaalia odontolyticus* was detected from peri-implant health sites. Firmicutes phylum was more dominant in the plaque sample from peri-implant health, while Actinobacteria, Bacteroidetes, Fusobacteria, and Proteobacteria were the relative abundant phyla in the plaque samples from peri-implant mucositis sites.

ANCOM-BC was also performed to assess differential abundance, we chose to present LEfSe results in the main text due to its broader identification of taxonomic patterns and more intuitive visualization. The results of the ANCOM-BC analysis are included in the Supporting Information (S5–S6 Table) to complement and validate the findings presented here.

## Discussion

This pilot study aimed to explore the correlation between pre-treatment periodontal diagnosis and peri-implant microbiome in edentulous patients with IROs. The pre-treatment periodontal diagnosis groups were determined based on distinct pre-extraction clinical measurements and radiographs. These groupings were intended to capture meaningful clinical contrasts by comparing the peri-implant microbiome across broader categories such as pre-extraction healthy gingiva versus localized or generalized periodontitis. In this study, heavy smokers were excluded due to evidence suggesting their significantly increased risk of implant failure, complications, and periodontal disease progression compared to non-smokers and light smokers. Light smokers were included to maintain an adequate sample size, allowing for more comprehensive analysis while minimizing the introduction of additional confounding factors [31–33]. Although it was hypothesized that more severe pre-treatment periodontitis would result in higher quantities and proportions of pathogens associated with peri-implant disease, our findings suggest a more complex relationship between these factors.

This study reported clinical parameters and subgingival microbial profiles of implants used as abutments for IROs. Although approximately 85% of patients had periodontitis prior to full-mouth extractions and IRO treatment, none of the bacterial species enriched in periodontitis [34] were detected as representative species from the peri-implant plaque. At the genus level, *Streptococcus*, *Rothia*, *Veillonella*, *Actinomyces*, and *Schaalia* were the top five bacteria present in most samples. Some of these genera have been reported as associated with periodontal health or peri-implant health [5,28]. In fact, only a few of bacterial species associated with periodontitis, include *Porphyromonas gingivalis*, *Filifactor alocis*, *Prevotella intermedia*, *Tannerella forsythia*, and *Treponema* sp., were detected from samples collected from one patient as non-dominant components. This specific patient had a pre-treatment diagnosis of Stage IV Grade C generalized periodontitis, and the implants were diagnosed with peri-implant mucositis. None of the other key periodontal pathogens were detectable from the rest of the plaque samples, suggesting that full-mouth extractions may effectively reduce or eliminate periodontal pathogens from the oral cavity. Our findings contrast with some previous studies that reported the periodontal pathogens could persist in the oral cavity of edentulous subjects who have had periodontitis after full-mouth tooth extraction [15,29], and the detection of certain periodontal pathogens, such as *Porphyromonas gingivalis*, *Actinobacillus actinomycetemcomitans*, and *Tannerella forsythia*, in subgingival plaques from nearly all patients who received IROs [12,13]. This discrepancy may be explained by the relatively short follow-up period in our study. Only three patients in our study had worn the IRO for longer than 5 years, close to 40% of patients had used the IRO less than a year. The initially pristine peri-implant pockets might not have enough time to become infected by periodontal pathogens that may exist in other niches in the oral cavity. Clinical findings from our study were consistent with microbiological findings, none of the implants included were diagnosed with peri-implantitis. Our study indicated that the severity of periodontitis prior to full-mouth tooth extraction does not significantly affect the number of periodontal pathogens detected in the peri-implant subgingival plaque of edentulous patients.

In contrast, significant differences in microbial diversity and composition were observed among pre-treatment diagnosis groups in this study. Higher microbial diversity was found in patients with generalized periodontitis compared to those with gingival health on a reduced periodontium prior to IRO treatment. These finding were consistent with some previous studies which have shown periodontitis sites were associated with a more diverse subgingival microbiota [30–32]. Beta diversity analysis further highlighted distinct clustering of peri-implant microbiomes based on pre-treatment periodontal status, IRO function time, and bone grafting. These patterns suggest that both biological and procedural factors may influence microbial diversity and composition.

This study identified specific taxa associated with different pre-treatment periodontal diagnoses. At the phylum level, some trends were observed when comparing the differential species associated with different pre-treatment diagnosis groups. Although our study did not find an association between pre-treatment periodontal diagnosis and current peri-implant status, the microbial differences observed may reflect residual microbial influences from the patient’s pre-treatment periodontal condition, even if short-term clinical outcomes remain unaffected. Future longitudinal studies will be necessary to elucidate the clinical consequences of these differences and to track microbial compositional shifts in these patients over time. In the current study, we observed a trend suggesting that peri-implant mucositis sites may have a more diverse subgingival microbiota, although this difference did not reach statistical significance. This observation is consistent with a recent systematic review [5] which suggested an increase of bacterial diversity with the progression of peri-implant disease. *Capnocytophaga gingivalis*, *Leptotrichia* sp. HMT 417, and *Schaalia odontolyticus* were almost exclusively present in peri-implant mucositis sites. There have been studies reporting that certain species of *Capnocytophaga* and *Leptotrichia* were associated with gingivitis, periodontitis, and peri-implantitis [35–38]. Our findings suggest that these species may also be associated with peri-implant mucositis. *Gemella sanguinis*, *Actinomyces oris*, *Streptococcus intermedius* and *Streptococcus oralis* were nearly not present at all in the peri-implant mucositis sites, indicating they might be associated with peri-implant health. *Gemella* is a genus of gram-positive facultatively anaerobic bacteria, and some *Gemella* species have been reported to be associated with periodontal health [39]. *Streptococcus oralis* has been well recognized as a probiotic species, that was associated with periodontal health [40,41]. *Actinomyces oris* plays a role in the early dental plaque formation and has been identified as enriched in subgingival plaque samples from patients with periodontal health [34,42]. Although *Streptococcus intermedius* has been reported to be associated with periodontal attachment loss [43] and clinical infections in other organs [44], the organism has been identified as a prevalent species in both periodontal plaque and peri-implant plaque in general [45,46]. Our study suggests that these three species may be associated with peri-implant health, particularly in implants used for supporting IROs.

Other factors beyond pre-treatment periodontal diagnosis may also influence the peri-implant subgingival microbiome. For instance, our study found that IRO function time and the use of bone grafts at the time of implant placement significantly impacted both bacterial species richness and the composition of bacterial communities in peri-implant subgingival plaque. Fewer observed bacterial species were detected when the IRO had been in function for more than one year, while Shannon and Simpson indices were slightly higher in this group, suggesting increased microbial diversity and evenness. These findings may indicate shifts in microbial composition over time rather than a true reduction in richness. Although this factor has not been previously explored in the literature, it warrants further investigation in future studies. Additionally, subgingival plaque samples from implant sites with bone graft material contained a greater variety of bacterial species. While gender and the number of remaining teeth prior to treatment may also influence bacterial species richness, these differences did not reach statistical significance. The number of remaining teeth prior to treatment was used as a grouping variable, with a threshold of fewer than 10 teeth chosen to reflect existing evidence suggesting that reduced dentition can influence the persistence of periodontal pathogens [47,48], which may, in turn, affect the peri-implant microbiome and health. This threshold was selected as a pragmatic cutoff point for this pilot study to explore potential correlations between pre-treatment oral conditions and peri-implant outcomes. Interestingly, male patients and patients who had more than 10 teeth prior to treatment tended to have fewer bacterial species detected in peri-implant plaque. These patterns suggest that both biological and procedural factors may influence peri-implant microbial diversity and composition. Nevertheless, evaluating the clinical significance resulting from these factors in relation to the variations in bacterial components within our study was challenging due to the limited sample size. The time interval between full mouth extraction and implant placement may also influence the subgingival microbiome. We did not consider this factor in our study because three patients in our sample had implants placed before undergoing full mouth extractions. Sixteen patients (64%) received implants less than 1 year after full mouth extraction, six patients received implants more than 1 year after the extractions. Patients were not given instructions to discontinue their regular oral hygiene practices before the collection of bacterial samples since suspension of oral hygiene practices may cause key bacterial shifts in subgingival plaques [49].

While this study was conducted using a robust methodology, it has some limitations. First, as a descriptive cross-sectional study that collected the pre-extraction data retrospectively, a key limitation is the lack of baseline microbiome data prior to full-mouth extraction, which prevents a direct evaluation of the relationship between initial periodontal dysbiosis and the peri-implant microbiome. Second, the sample size was small, limiting the study’s power and generalizability. Additionally, the confounding effects of variables were not evaluated, and the functional period of the IRO after implant placement was relatively short. While beta diversity analysis revealed differences in microbial composition based on pre-treatment periodontal diagnosis, IRO function time, and bone grafting, we were unable to perform a multivariate analysis due to the limited sample size in this study. Another potential confounder is smoking status; although we reported the smoking status by gender, the small number of smokers limited our ability to evaluate its influence on microbial composition, particularly in relation to observed gender-based differences in beta diversity. The average age of patients in this study was 71 years, with the youngest being 59 years old. Studies have shown that there are age-related changes in the immune system that regulate inflammation, and the elderly population may react differently to periodontal, and peri-implant microbial challenges compared to the younger population [47–49]. The time interval between full mouth extraction and implant placement may also influence the subgingival microbiome. We did not consider this factor in our study because three patients in our sample had implants placed before undergoing full mouth extractions. Sixteen patients (64%) received implants less than 1 year after full mouth extraction, six patients received implants more than 1 year after the extractions. Patients were not given instructions to discontinue their regular oral hygiene practices before the collection of bacterial samples since suspension of oral hygiene practices may cause key bacterial shifts in subgingival plaques [49]. However, different oral hygiene practices may affect the subgingival microbiome. Another limitation of this research is the grouping of periodontal diagnoses, which, while designed to balance clinical relevance and allow meaningful comparisons, may not provide the level of detail needed for a more nuanced analysis. It is also important to note that clinical gingival health on a reduced periodontium does not necessarily indicate a history of periodontitis; patients in this category were classified based on clinical and radiographic records, without definitive documentation of past periodontal disease. Additionally, due to the limited sample size and absence of peri-implantitis cases in this pilot study, we did not perform predictive modeling analyses. As a descriptive study, this work serves as a foundation for future hypothesis-driven research, highlighting the need for multivariate analyses to account for potential confounders. Well-designed longitudinal or experimental studies with larger sample sizes and more balanced cohorts will be essential for gaining deeper insights into these relationships.

## Conclusion

This study suggests that while pre-treatment periodontal diagnosis may impact the peri-implant subgingival microbiome in patients who received implant-retained overdentures, it does not appear to be directly associated with clinical peri-implant outcomes. Bacterial species enriched in periodontitis may decrease to undetectable levels in the peri-implant plaque after full mouth extraction. Additionally, factors such as the functional lifespan of the implant-retained overdenture and the use of bone grafts during implant placement may also impact the peri-implant microbiome. These findings highlight the complex interaction between host factors, procedural variables, and microbial dynamics in shaping peri-implant health.

## Supporting information

S1 Table ASV Read Count Table by Sample. (XLSX)

S2 Fig Alpha Rarefaction Curve – Shannon Diversity. (PDF)

S3 Fig Alpha Rarefaction Curve – Simpson Diversity. (PDF)

S4 Fig Alpha Rarefaction Curve – Observed Features. (PDF)

S5 Table Peri-implant diagnosis_ANCOMBC. (XLSX)

S6 Table Pre-treatment periodontal diagnosis_ANCOMBC. (XLSX)
