# Supplementary material for: Correlation between pre-extraction periodontal diagnosis and peri-implant microbiome for patients treated with implant-retained overdenture – A retrospective cohort pilot study
Source: PLoS One. 2025 Jul 14;20(7):e0325711. doi: 10.1371/journal.pone.0325711 (PMC12258591; doi:10.1371/journal.pone.0325711)

Alpha rarefaction

Download CSV

Metric

shannon

Sample Metadata Column

SampleName

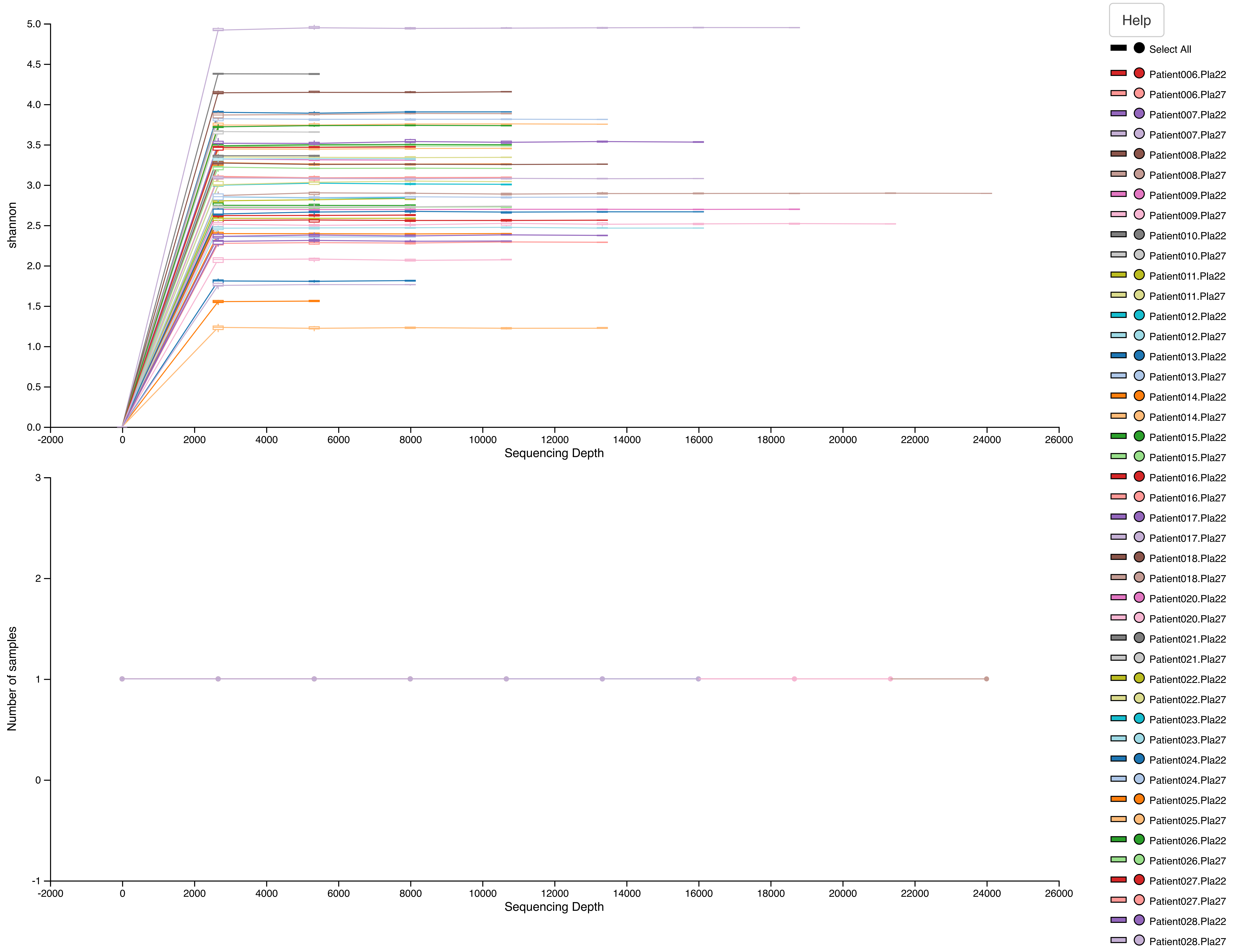

Supplement: S2 Fig — (PDF) [file pone.0325711.s002.pdf]
